# Metabolic Cycles Are Linked to the Cardiovascular Diurnal Rhythm in Rats with Essential Hypertension

**DOI:** 10.1371/journal.pone.0017339

**Published:** 2011-02-22

**Authors:** He Cui, Akira Kohsaka, Hidefumi Waki, Mohammad E. R. Bhuiyan, Sabine S. Gouraud, Masanobu Maeda

**Affiliations:** Department of Physiology, Wakayama Medical University School of Medicine, Wakayama, Japan; Pennsylvania State University, United States of America

## Abstract

**Background:**

The loss of diurnal rhythm in blood pressure (BP) is an important predictor of end-organ damage in hypertensive and diabetic patients. Recent evidence has suggested that two major physiological circadian rhythms, the metabolic and cardiovascular rhythms, are subject to regulation by overlapping molecular pathways, indicating that dysregulation of metabolic cycles could desynchronize the normal diurnal rhythm of BP with the daily light/dark cycle. However, little is known about the impact of changes in metabolic cycles on BP diurnal rhythm.

**Methodology/Principal Findings:**

To test the hypothesis that feeding-fasting cycles could affect the diurnal pattern of BP, we used spontaneously hypertensive rats (SHR) which develop essential hypertension with disrupted diurnal BP rhythms and examined whether abnormal BP rhythms in SHR were caused by alteration in the daily feeding rhythm. We found that SHR exhibit attenuated feeding rhythm which accompanies disrupted rhythms in metabolic gene expression not only in metabolic tissues but also in cardiovascular tissues. More importantly, the correction of abnormal feeding rhythms in SHR restored the daily BP rhythm and was accompanied by changes in the timing of expression of key circadian and metabolic genes in cardiovascular tissues.

**Conclusions/Significance:**

These results indicate that the metabolic cycle is an important determinant of the cardiovascular diurnal rhythm and that disrupted BP rhythms in hypertensive patients can be normalized by manipulating feeding cycles.

## Introduction

In humans, diurnal variation in cardiovascular function, including the regulation of blood pressure (BP) and heart rate (HR), is among the best recognized physiological rhythms [1], [2]. One benefit of the cardiovascular diurnal rhythm is that it enables timely modulation of blood flow to organs according to their daily demands across the sleep-wake cycle. Dysregulation of the diurnal rhythm of BP may cause pressure overload within organs during the sleep cycle, facilitating the malfunctioning of major organs, including the heart, kidneys, brain, and eyes, in which the blood vessels are highly susceptible to damage caused by excessive BP. Consistent with this notion are clinical observations that an insufficient reduction in BP during sleep is associated with an increased risk of heart failure, stroke, and glomerular dysfunction in hypertensive and diabetic patients [3]–[5]. Furthermore, recent evidence in rodents suggests that disrupted BP rhythm impairs vascular endothelial function, further implicating the cardiovascular diurnal rhythm in the maintenance of human health [6].

The cardiovascular diurnal rhythm, like many other physiological and biological rhythms, is programmed by the circadian system. In this system, the master pacemaker in the suprachiasmatic nucleus (SCN) of the anterior hypothalamus is entrained to the daily light/dark (LD) cycle and subsequently transmits synchronizing signals to local clocks in peripheral tissues [7]. On the molecular level, it has become clear that cardiovascular tissues, including the heart and aorta, possess local clocks whose core molecular structures are similar to that of the master pacemaker in the SCN [8], [9]. The molecular clock is composed of interconnected feedback loops of gene transcription and translation in which the heterodimeric CLOCK/BMAL1 transcription factor complex activates the transcription of repressor clock genes such as the *Period* genes (*Per1* and *Per2*) and *Cryptochrome* genes (*Cry1* and *Cry2*). These protein products in turn inhibit CLOCK/BMAL1 transcriptional activity, thereby reducing their own transcription [7]. Recent studies showing that the diurnal variation in BP is disrupted in *Clock* mutant, *Bmal1* knockout, and *Cry1/Cry2* double knockout animals indicate that genetic components of the circadian system are required to generate the diurnal BP rhythm [10], [11].

While the molecular clocks in both the SCN and the peripheral tissues are self-sustained and cell autonomous [12], they are entrained to external cues to generate the appropriate 24 hr rhythms. Among these external cues, light and food are two strong stimuli that can entrain the molecular clock [13], [14]. Light entrainment is the dominant cue for the SCN clock, but the timing of food intake also appears to be important in resetting the phase of clock gene expression in peripheral tissues, including the heart [15]–[18], indicating that metabolic signaling from ingested food might be a driving force for the generation of the cardiovascular diurnal rhythm. Very little is known about how metabolic signals communicate with local clocks in cardiovascular tissue; however, studies employing whole-transcriptome profiling and gene targeting in cardiovascular tissue have revealed a possible molecular link between the metabolic cycle and the cardiovascular rhythm. Microarray studies have shown that the expression of metabolic genes involved in carbohydrate and fat metabolism displays diurnal variation not only in metabolic tissues but also in cardiovascular tissues [19]–[21]. Furthermore, recent studies have shown that mice with a blood vessel-specific deletion of peroxisome proliferator-activated receptor-γ (PPARγ), a nuclear receptor transcription factor that both acts as a lipid sensor and interacts with the molecular clock, have disrupted diurnal BP rhythms. This indicates that the regulation of the cardiovascular diurnal rhythm is tightly coupled with metabolic cycles at the molecular level [22].

In the current study, we hypothesized that dysregulation of the daily BP rhythm in mammals may be caused by altered metabolic cycles. We used spontaneously hypertensive rats (SHR), which develop essential hypertension with disrupted BP rhythm, to demonstrate that changes in metabolic cycles can account for the dysregulation of the cardiovascular diurnal rhythm.

## Results

### Impaired Diurnal Control of Central Feeding Circuits in SHR

**Figure 1:**
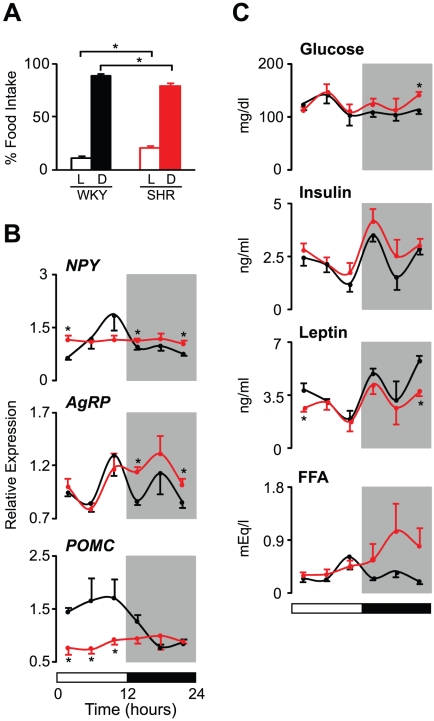
Diurnal control of central feeding circuits is impaired in SHR. (A) The diurnal rhythm of food intake in WKY (black bars, n = 6) and SHR (red bars, n = 6). Food intake was measured during 12 hr light (L) and dark (D) periods. Average food intake during each period is expressed as a percentage of total food intake. (B and C) Diurnal variation in the mRNA expression levels of neuropeptides (*NPY*, *AgRP*, and *POMC*) in the mediobasal hypothalamus (B) and in blood metabolic parameters (glucose, insulin, leptin, and FFA) (C). Tissues and blood were collected every 4 hr from WKY (black lines, n = 6 per time point) and SHR (red lines, n = 6 per time point). Values for mRNA expression are shown as relative expression levels normalized to *β-actin*. The 12∶12 LD cycle is indicated by the bars at the bottom of the figure. All data are expressed as means ± SEM. *p<0.05.

To demonstrate that SHR have abnormal metabolic cycles, we first characterized the diurnal rhythms of feeding behavior, hypothalamic neuropeptide expression, and circulating hormones and nutrients in these animals (Figure 1). Both SHR and control (normotensive) Wistar-Kyoto rats (WKY) were acclimated to 12∶12 light:dark (LD) conditions for a week; food intake was then measured during the light and dark periods. WKY showed a clear diurnal rhythm of feeding behavior, with only 11% of food intake occurring during the rest (light) period, whereas 20% of food intake occurred during the rest period in SHR (Figure 1A). The attenuation in the diurnal variation of feeding behavior in SHR was caused by significant increase in food intake during the light period and by a mild decrease during the dark period (Table S1). Interestingly, hyperphagia during the light period resulted in a significant increase in total food intake over 24 hrs in SHR, although the SHR were leaner than the WKY (Table S1).

We next examined diurnal rhythms in the levels of transcripts encoding hypothalamic neuropeptides that affect feeding behavior and energy balance (Figure 1B). These included selected orexigenic [neuropeptide Y (NPY) and agouti-related protein (AgRP)] as well as anorexigenic [pro-opiomelanocortin (POMC)] neuropeptides expressed in the mediobasal hypothalamus (MBH). As expected, the expression patterns of neuropeptides across the LD cycle corresponded to the feeding behavior pattern in control animals (Figure 1B). In particular, the peak expression levels of *NPY* and *AgRP* were observed just before the onset of feeding behavior in WKY (Figure 1B). The expression of *POMC* in WKY was sustained at a higher level during the light period, when appetite is normally suppressed (Figure 1B). In contrast, SHR exhibited no temporal change in the expression levels of *NPY* and *POMC* (Figure 1B). An altered diurnal rhythm of *AgRP* levels was also found in SHR, with the peak expression shifted toward the dark period (Figure 1B). Notably, SHR exhibited significantly higher levels of the orexigenic neuropeptides *NPY* and *AgRP* at several time points of the LD cycle as well as markedly lower levels of the anorexigenic neuropeptide *POMC* at all time points during the light period (Figure 1B).

Since circulating nutrients and hormones secreted from metabolic tissues can directly affect the expression levels of hypothalamic neuropeptides [23]–[25], we also analyzed 24 hr profiles of glucose, insulin, leptin, and free fatty acids (FFA) in serum (Figure 1C). All four of these metabolic parameters displayed diurnal variation in both WKY and SHR (Figure 1C). However, the absolute levels and/or specific profile patterns differed between these two strains (Figure 1C). Serum glucose levels in SHR were significantly increased at the end of the dark period compared to those observed in WKY (Figure 1C). The overall levels of insulin also tended to be higher at virtually all time points of the LD cycle in SHR, although this difference was not statistically significant (Figure 1C and Table S2). In contrast to glucose and insulin, the overall levels of leptin were significantly reduced in SHR, particularly during the dark-to-light transition period (Figure 1C and Table S2). Interestingly, while the diurnal patterns of glucose, insulin, and leptin levels were not different between WKY and SHR, an altered diurnal rhythm of FFA levels was observed in SHR (Figure 1C). In particular, while the peak FFA level in WKY was observed at the end of the light period, the peak in SHR occurred during the dark period (Figure 1C). We also observed a significant increase in overall FFA levels in SHR (Table S2). Collectively, these findings reveal that SHR have disordered diurnal control of feeding circuits at the molecular and behavioral levels.

### Disrupted Diurnal Regulation of Energy Metabolism in Liver and Fat in SHR

**Figure 2:**
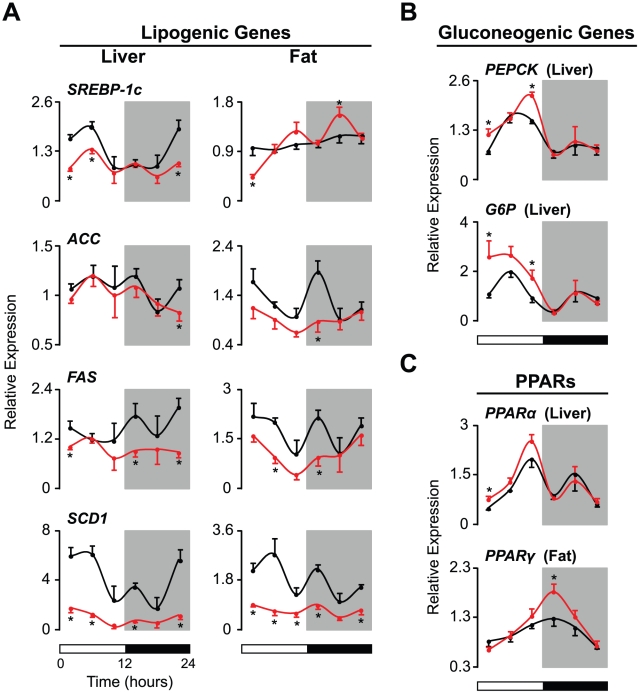
Diurnal control of peripheral energy metabolism is disrupted in SHR. Animals were maintained on a 12∶12 LD cycle (indicated by the bars at the bottom) and fed ad libitum. Liver and visceral fat tissues were collected every 4 hr from WKY (black lines, n = 6 per time point) and SHR (red lines, n = 6 per time point). (A) Diurnal variation in the mRNA expression levels of lipogenic genes in the liver and visceral fat. (B) Diurnal variation in the transcript levels of gluconeogenic genes in the liver. (C) Diurnal expression patterns of *PPARα* in the liver and *PPARγ* in visceral fat. Transcript levels were determined by real-time PCR. Values are shown as relative expression levels normalized to *β-actin*. Results are expressed as means ± SEM. *p<0.05.

We next investigated whether the diurnal regulation of energy metabolism in peripheral tissues was also altered in SHR (Figure 2). Since the disrupted FFA rhythm observed in SHR could be induced by changes in the expression of genes controlling lipid metabolism, we first examined the 24 hr expression profiles of lipogenic genes in liver and fat tissues. We investigated the sterol regulatory element-binding protein 1c (SREBP-1c) transcript because SREBP-1c is a key transcription factor that regulates fatty acids and triglyceride synthesis depending on whether the animal is in a fed or fasted state. We also analyzed the diurnal expression of acetyl-CoA carboxylase (ACC), fatty acid synthase (FAS), and stearoyl-CoA desaturase-1 (SCD1) transcripts because the expression levels of these genes are directly regulated by SREBP-1c. In WKY, levels of *SREBP-1c* displayed profound diurnal variation in the liver, peaking during the dark-to-light transition period (Figure 2A, left panel). Similar time-dependent variation was observed for the expression levels of *ACC*, *FAS*, and *SCD1* in the liver of WKY (Figure 2A, left panel). Interestingly, however, the diurnal variation of the hepatic expression of *SREBP-1c*, *ACC*, *FAS*, and *SCD1* was attenuated in SHR compared to WKY. In addition, the levels of all four of these lipogenic genes were significantly decreased in liver tissue from SHR (Figure 2A, left panel).

In fat tissue, WKY also exhibited time-dependent variation in *SREBP-1c*, *ACC*, *FAS*, and *SCD1* levels, although the patterns of expression were different from those observed in the liver (Figure 2A). While the overall levels of *SREBP1c* in fat tissue did not differ between WKY and SHR, the levels of *ACC*, *FAS*, and *SCD1* in fat tissue were decreased in SHR compared to WKY, resulting in attenuated diurnal variation in the levels of each of these transcripts (Figure 2A, right panel).

Since blood glucose and insulin levels were increased in SHR, we also analyzed the temporal regulation of genes involved in hepatic gluconeogenesis (Figure 2B). We studied phosphoenolpyruvate carboxykinase (PEPCK) and glucose-6-phosphatase (G6P) mRNA expression in the liver because an increase in the expression of these genes is associated with hepatic insulin resistance. In both WKY and SHR, the levels of *PEPCK* and *G6P* varied across the 24 hr LD cycle, with peaks during the light period (Figure 2B). Although the diurnal patterns of *PEPCK* and *G6P* expression did not differ between the two strains, the levels of these gluconeogenic genes were increased at the beginning and end of the 12 hr light phase in SHR (Figure 2B). These increases were consistent with the mild insulin resistance observed in SHR (Figure 1C and Figure 2B).

We also examined the mRNA levels of *PPARα* in the liver and *PPARγ* in fat because these proteins have been shown to directly regulate both clock gene function and lipid metabolism (Figure 2C) [22], [26]. Both WKY and SHR displayed diurnal variation in the levels of *PPARα* in the liver and *PPARγ* in fat (Figure 2C). In particular, we observed a peak in *PPARα* expression at the end of the light period and a peak in *PPARγ* expression at the beginning of the dark period in both strains (Figure 2C). While the diurnal patterns of expression of *PPARα* and *PPARγ* were similar in WKY and SHR, increased expression levels were observed in tissues from SHR (Figure 2C).

### Altered Clock Gene Expression in Metabolic Tissues of SHR

**Figure 3:**
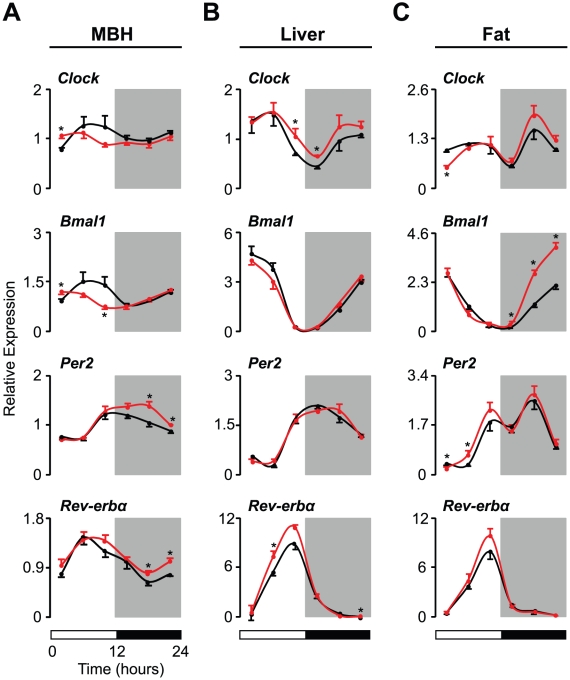
Clock gene expression is altered in metabolic tissues of SHR. [Note that the data in this figure were derived from the same animals represented in Figure 1 (the mediobasal hypothalamus, MBH) and Figure 2 (liver and visceral fat).] Transcript levels of core clock genes (*Clock*, *Bmal1*, *Per2*, and *Rev-erbα*) in (A) the MBH, (B) the liver, and (C) visceral fat were determined by real-time PCR in WKY (black lines, n = 6 per time point) and SHR (red lines, n = 6 per time point). Values are displayed as relative expression levels normalized to *β-actin*. White and black bars below the figures represent the 12∶12 LD cycle. All data represent means ± SEM. *p<0.05.

To further explore whether the disrupted rhythms of energy metabolism observed in SHR are due to changes in clock gene expression in metabolic tissues, we analyzed the diurnal patterns of the mRNA expression of core clock genes, including *Clock*, *Bmal1*, *Per2* and *Rev-erbα,* within the MBH, liver, and fat (Figure 3). In the MBH of WKY, all four of these clock genes displayed small but clear diurnal variations in expression levels (Figure 3A). In SHR, however, the expression of *Clock* and *Bmal1* within the MBH was increased at the beginning of the light phase and decreased at the end of light phase, resulting in a phase shift of the diurnal expression of these clock genes (Figure 3A). We also observed a shift in the phase of *Per2* expression levels in the MBH of SHR (Figure 3A). In contrast to *Clock*, *Bmal1*, and *Per2*, the timing of *Rev-erbα* expression did not differ between WKY and SHR, although the expression levels of *Rev-erbα* were significantly higher in SHR from the middle to the end of the dark period (Figure 3A).

In contrast to the MBH, the diurnal expression patterns of *Clock*, *Bmal1*, *Per2*, and *Rev-erbα* in both liver and fat tissues were similar in WKY and SHR (Figures 3B and 3C), but the expression levels of all four of these clock genes differed. In particular, increased levels of *Clock* transcripts in the liver of SHR were observed during the light-to-dark transition period, whereas *Clock* levels in fat at the beginning of the light period were decreased in SHR compared to WKY (Figures 3B and 3C). SHR also displayed increased levels of *Bmal1* in fat (Figure 3C). In addition, SHR showed generally increased levels of *Per2* in fat and *Rev-erbα* in the liver during the light period (Figures 3B and 3C). Overall, we observed broad changes in the diurnal regulation of both metabolic and clock genes in tissues involved in energy metabolism in SHR.

### Restricted Feeding Restores Diurnal Rhythms of BP and HR in SHR

To test our hypothesis that alteration of metabolic cycles affects the diurnal cardiovascular rhythm, we first characterized the cardiovascular phenotypes of WKY and SHR using radiotelemetry. We examined average levels of BP and HR during light and dark periods in WKY and SHR (Table S3). Consistent with previous findings, SHR displayed significantly higher levels of systolic and diastolic BP during both the light and the dark periods (Table S3). In contrast, HR was significantly reduced throughout the 12∶12 LD cycles in SHR compared to WKY (Table S3).

**Figure 4:**
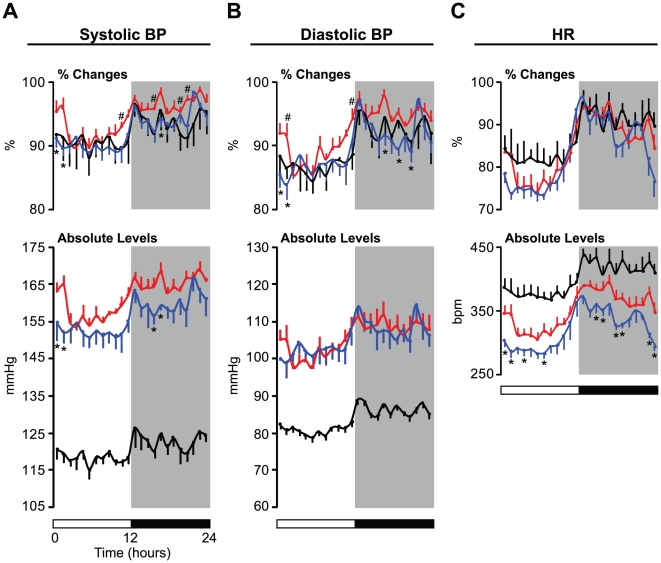
Effects of restricted feeding on diurnal rhythms and levels of BP and HR in SHR. Blood pressure (BP) and heart rate (HR) were telemetrically monitored in freely moving animals maintained on a 12∶12 LD cycle (indicated by the bars at the bottom). Data were collected for the first 5 min of every 60 min; the value at each time point represents an average of values taken over 2 consecutive days. (A) Systolic and (B) diastolic BP and (C) HR data are expressed as the mean (± SEM) of the percent change from the highest value in each individual animal (top panels) and as absolute levels (bottom panels). After BP and HR were recorded in WKY (black lines, n = 6) and SHR (red lines, n = 6) fed ad libitum, the same animals were exposed to a restricted feeding regimen in which food was provided only during the active (dark) period. BP and HR were monitored again during the fourth and fifth days of the RF regimen. Only RF data from SHR (blue lines, n = 6) are shown for visual clarity. The value shown at each time point represents the mean ± SEM. #, SHR versus WKY; *, SHR ad libitum versus RF; p<0.05.

We also performed a detailed analysis of BP and HR profiles over 24 hours in WKY and SHR (Figure 4). These are shown as percent changes in BP and HR to visually clarify differences in the diurnal variation of these cardiovascular parameters between experimental groups (Figures 4, top panels) as well as in the absolute levels of these cardiovascular parameters (Figure 4, bottom panels). WKY exhibited a robust diurnal rhythm of both systolic and diastolic BP, with higher levels during the dark period and persistently lower levels throughout the light period (Figures 4A and 4B). Interestingly, although SHR displayed diurnal rhythms of systolic and diastolic BP, the patterns of diurnal variation were different from those observed in WKY (Figures 4A and 4B, top panels). In particular, the distinct BP patterns in SHR were characterized by (1) a more gradual reduction after the beginning of the light period, (2) earlier increase prior to the active (dark) period, and (3) sustained, increased levels during the mid-dark period, while WKY exhibited relatively lower levels in the dark period (Figures 4A and 4B). We also observed a clear diurnal HR pattern in WKY (Figure 4C). In parallel with the changes in the BP rhythm, SHR exhibited a similar alteration in the diurnal rhythm of HR, although the change was not statistically significant (Figure 4C).

To further explore whether the changes in cardiovascular diurnal rhythm observed in SHR are due to changes in feeding cycles, we exposed SHR to a restricted feeding (RF) regimen in which food was available only during the dark period and monitored BP and HR (Figure 4). Interestingly, the increased percent changes in systolic and diastolic BP at the beginning of the light period were significantly reduced in SHR exposed to this regimen (Figures 4A and 4B, top panels). Similar hypotensive effects of RF on percent changes in systolic and diastolic BP at the end of the light period were also observed in SHR, although these were not statistically significant (Figures 4A and 4B, top panels). Furthermore, increased percent changes in the levels of systolic and diastolic BP during the mid-dark period in SHR were also markedly suppressed by RF (Figures 4A and 4B). In addition, we were surprised to find that the overall levels of systolic BP were decreased in SHR exposed to RF, although we did not observe such effects of RF on diastolic BP levels (Figures 4A and 4B, bottom panels). We also analyzed the effects of RF on diurnal variation in the HR of SHR. Although the difference was not statistically significant, increased levels of HR at the beginning of the light period were somewhat suppressed by RF (Figure 4C, top panel). In addition, SHR exposed to RF displayed lower overall HR during both the light and the dark periods (Figure 4C, bottom panel).

To ensure that the RF regimen did not induce a negative energy balance, which might on its own affect the regulation of cardiovascular function, we examined food intake and body weight in SHR fed ad libitum and SHR under RF conditions (Table S4). Neither total food intake over 24 hrs nor body weight differed between feeding paradigms (ad libitum versus RF) in SHR (Table S4). Taken together, these results demonstrate that feeding cycles affect the cardiovascular diurnal rhythm in SHR.

### Restricted Feeding Restores the Diurnal Rhythms of Clock and Metabolic Gene Expression in Cardiovascular Tissues in SHR

**Figure 5:**
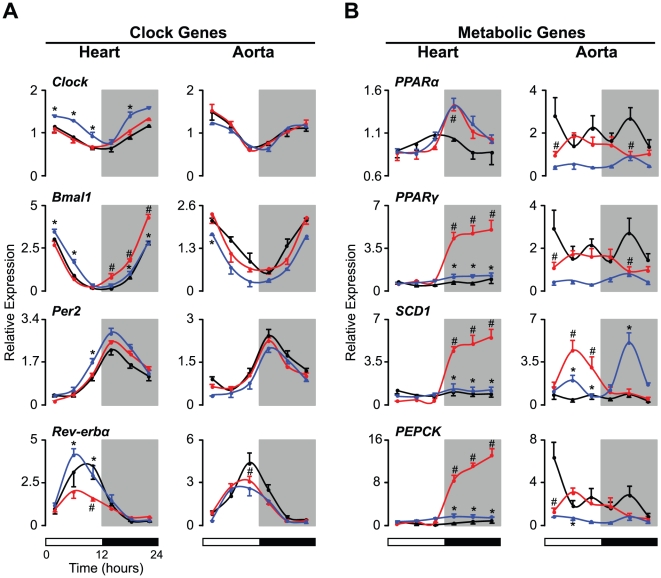
Effects of restricted feeding on clock and metabolic gene expression in cardiovascular tissues of SHR. The levels of transcripts from (A) clock and (B) metabolic genes in the heart and aorta were determined by real time PCR. Animals were either fed ad libitum or exposed to an RF regimen, in which food was provided exclusively during the dark period, for 5 consecutive days. Heart and aorta tissues were harvested every 4 hr on the fifth day under these feeding conditions. For visual clarity, only data from the ad libitum-fed WKY (black lines, n = 4 per time point) and SHR (red lines, n = 4 per time point) and RF-fed SHR (blue lines, n = 4 per time point) are shown. Values for mRNA expression are displayed as relative expression levels normalized to *β-actin*. The 12∶12 LD cycle is indicated by the bars at the bottom of the figure. All data are expressed as means ± SEM. #, SHR versus WKY; *, SHR fed ad libitum versus RF; p<0.05.

To examine the molecular mechanisms that link feeding cycles with the diurnal cardiovascular rhythm, we analyzed the expression of clock and metabolic genes within the heart and aorta (Figure 5). Consistent with previous findings, the levels of *Clock*, *Bmal1*, *Per2*, and *Rev-erbα* expression displayed robust diurnal variation in both the heart and the aorta in WKY (Figure 5A). In SHR, all four of these clock genes exhibited expression rhythms similar to those observed in WKY; however, *Bmal1* and *Rev-erbα* levels were altered in SHR (Figure 5A). In particular, *Bmal1* expression in the heart was significantly increased during the dark period, and the peak levels of Rev-erbα expression in both heart and aortic tissue were significantly reduced (Figure 5A). Surprisingly, however, the expression of these genes was restored to the levels observed in WKY upon exposure to an RF regimen (Figure 5A, left panel). We also investigated the effects of RF on the expression of *Clock* and *Per2* in the heart of SHR (Figure 5A, left panel). In particular, the overall levels of *Clock* expression were increased in SHR exposed to RF (Figure 5A, left panel). RF also caused an increase in *Per2* expression at the end of the light period in SHR (Figure 5A, left panel). In contrast to the broad effects of RF on clock genes in the heart, this feeding regimen affected a decrease in *Bmal1* only at the beginning of the light period in the aorta (Figure 5A, right panel).

Because genes encoding CRY1 and CRY2 have recently been shown to play an important role in the development of salt-induced hypertension [27], we examined the expression levels of these clock genes (Figure S1). The levels of *Cry1* displayed clear diurnal variation in both the heart and the aorta in WKY (Figure S1). SHR exhibited similar diurnal rhythms for *Cry1* in both tissues, but levels in the aorta were decreased at the beginning of the light phase regardless of feeding condition (Figure S1). In heart and aortic tissues, *Cry2* also showed minor diurnal changes in WKY (Figure S1). However, *Cry2* expression in SHR heart tissue displayed an augmented diurnal rhythm that could be partially restored by RF (Figure S1). Interestingly, in parallel with the changes in *Cry1* expression in the aorta, levels of *Cry2* in the aorta were also reduced at the beginning of the light period in SHR (Figure S1).

We also studied PPARα and PPARγ transcription in cardiovascular tissues because these PPARs are abundantly expressed not only in metabolic tissues, but also in cardiovascular tissues (Figure 5B). In WKY, levels of *PPARα* displayed diurnal variation in both the heart and the aorta, although the patterns of expression were distinct between the two tissues (Figure 5B). SHR also exhibited time-dependent variation in *PPARα* levels in the heart and aorta; however, in contrast to the liver, the phase of peak *PPARα* expression differed between the two strains (Figure 2C and Figure 5B). Similarly, we observed strain-dependent expression patterns in *PPARγ* levels in both heart and aortic tissues (Figure 5B). Both the diurnal patterns and the expression levels of the *PPARs* differed between WKY and SHR. Elevated *PPARα* and *PPARγ* levels in the heart were observed during the dark period, although the expression of both *PPARs* in the aorta was reduced during the early light phase and the mid-dark period in SHR (Figure 5B). In heart tissue, while a RF regimen did not alter *PPARα* expression, it was able to completely restore normal *PPARγ* expression levels during the dark period (Figure 5B). In addition, the overall *PPARα* and *PPARγ* levels in the aorta were lower in SHR exposed to RF than in those fed ad libitum, although this difference was not significant (Figure 5B).

Since cardiovascular tissues constantly require energy throughout the sleep/wake cycle, we examined the expression levels of genes that can sense changes in energy balance. We studied *SCD1* because recent studies suggest that this gene plays an important role in glucose and lipid metabolism in the heart (Figure 5B) [28]. We also analyzed *PEPCK*, which is known to cycle in the aorta (Figure 5B) [21]. In heart tissue, neither *SCD1* nor *PEPCK* displayed diurnal variation in expression in WKY (Figure 5B), while increased levels of both *SCD1* and *PEPCK* during the dark period were observed in SHR (Figure 5B). Interestingly, however, a RF regimen completely abolished these changes such that gene expression was reduced to the levels of WKY (Figure 5B).

Although we did not observe diurnal variation in *SCD1* expression in the aorta of WKY, *PEPCK* RNA levels displayed a diurnal rhythm of expression, peaking during the first part of the light period (Figure 5B). However, the peak expression levels of *PEPCK* were significantly attenuated in the aorta of SHR (Figure 5B). In contrast to *PEPCK*, *SCD1* expression in the aorta was significantly higher in SHR than in WKY; however, the increases occurred during the light period and were only partially restored by RF, in contrast to its expression in heart tissue (Figure 5B). In addition, when compared to SHR fed ad libitum, SHR on a RF regimen displayed elevated levels of *SCD1* in the aorta during the dark period (Figure 5B).

Circulating FFA directly affects the expression of metabolic genes in cardiovascular tissues [29]. We therefore analyzed the effects of RF on the diurnal pattern of FFA levels in SHR (Figure S2). When SHR were placed on a RF schedule, these animals displayed restored rhythms as well as decreased levels of FFA levels across the 24 hr LD cycle (Figure S2). Collectively, these findings suggest that metabolic cycles originating from feeding behavior determine temporal changes in the expression of both clock and metabolic genes in cardiovascular tissues.

### Effects of Restricted Feeding on the Diurnal Rhythms of Serum Corticosterone and Urine Norepinephrine in SHR

**Figure 6:**
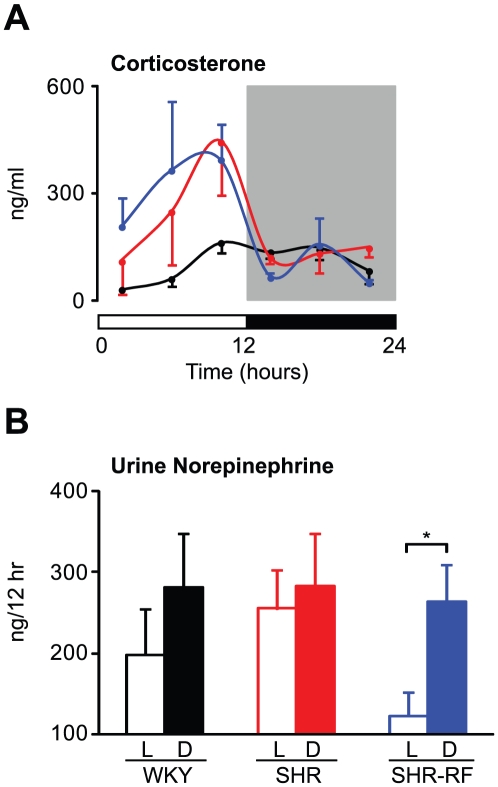
Effects of restricted feeding on diurnal rhythms in glucocorticoid and catecholamine levels in SHR. Animals maintained under a 12∶12 LD cycle were fed ad libitum or maintained under restricted feeding (RF) conditions for 5 consecutive days. Under the RF condition, food was available only during the active (dark) period. (A) Serum corticosterone levels were analyzed by ELISA. On the fifth day of the feeding regimen, blood was collected every 4 hr from the free-fed WKY (black lines, n = 4 per time point) and SHR (red lines, n = 4 per time point) and the RF-fed SHR (blues lines, n = 4 per time point). (B) Urine norepinephrine levels were determined for the light (L) and dark (D) periods. On the fourth and fifth days of the RF regimen, urine was collected from the free-fed WKY (black bars, n = 4) and the SHR (red bars, n = 4) as well as the RF-fed SHR (blue bars, n = 4) in vials containing 100 µl 6 M HCl. The results shown represent averages over 2 consecutive days. All values are expressed as means ± SEM. *p<0.05.

We also evaluated diurnal variation in serum corticosterone levels because the dysregulation of rhythms in both metabolic and cardiovascular function observed in SHR could be explained by changes in glucocorticoid regulation (Figure 6A). Consistent with previous findings, WKY animals displayed a clear diurnal rhythm in corticosterone levels with a peak just before the active (dark) period (Figure 6A). In SHR, we observed a similar time-dependent cycling of corticosterone, but the levels of the hormone differed between WKY and SHR (Figure 6A). In particular, increased levels of corticosterone during the light period were observed in SHR compared to WKY, although this difference did not reach statistical significance (Figure 6A). In contrast to the broad effects of RF on serum FFA and gene expression levels, neither diurnal variation nor the levels of corticosterone were altered in SHR exposed to RF (Figure 6A).

Since sympathetic nerve activity also affects cardiovascular and metabolic processes, we analyzed urine norepinephrine levels; norepinephrine is the predominant catecholamine synthesized in the peripheral sympathetic nerves (Figure 6B). While not statistically significant, WKY exhibited a slight diurnal variation in urine norepinephrine levels (Figure 6B). In SHR, we observed attenuated diurnal variation in urine norepinephrine levels (Figure 6B). Interestingly, RF provoked a clear diurnal rhythm in the norepinephrine levels in SHR (Figure 6B).

## Discussion

### The Daily Cycle of Energy Metabolism Drives the Diurnal Cardiovascular Rhythm

When clock genes were identified in mammals, it became clear that regulation of these genes is tightly coupled to energy metabolism as well as to the photoperiod. Perhaps most striking was the demonstration that feeding rodents exclusively during the rest (light) period, when they normally sleep, inverts the phase of diurnal variation in the clock gene's expression in metabolic tissues, including the liver and fat [30], [31]. A similar phase resetting of clock genes has also been observed in the heart [15], [17], [18], suggesting that, even in cardiovascular tissues, clock genes sense metabolic cycles. Our observation that changes in feeding cycles affect diurnal variation in BP and HR indicates that the response to changes in metabolic cycles is not limited to the level of gene expression (i.e., clock and metabolic genes) but reaches the level of physiological functions (e.g., BP and HR). Although the difference between the feeding rhythms of SHR exposed to RF and ad libitum feeding was subtle (i.e., 100% versus 80% food intake during the active period), this small alteration in feeding cycles was sufficient to affect the cardiovascular diurnal rhythm. Recent studies showing that the *db/db* mouse, an animal model of obesity and diabetes, exhibits a disrupted diurnal rhythm of BP also suggest that the cardiovascular diurnal rhythm is sensitive to changes in energy metabolism [32], [33].

We also examined the 24 hr profile of locomotor activity as measured by infrared beam crossing (data not shown) because voluntary locomotor activity is closely associated with BP and HR. However, we observed no correlation between activity change and BP (or HR) change. For example, SHR exhibited increases in BP levels at the beginning and the end of the light period, but we did not observe corresponding increases in locomotor activity at identical time periods. In addition, despite the significant effects of the RF regimen on BP and HR rhythms in SHR, RF did not influence the diurnal variation of locomotor activity in these animals. These results suggest that the alterations of diurnal BP and HR rhythms observed in SHR were not caused by a change in locomotor activity.

### Complex Interplay between Circadian and Metabolic Genes in the Cardiovascular System

At the molecular level, we observed that metabolic cycles directly or indirectly affect the temporal patterns and levels of expression of both circadian and metabolic genes in cardiovascular tissues. These results indicate that the diurnal rhythms in BP and HR are coordinated by complex temporal networks of circadian and metabolic genes in cardiovascular tissues and raise several interesting possibilities. First, it is possible that feeding cycles affect diurnal variation primarily via clock gene expression, which then determines metabolic gene expression. In fact, the CLOCK/BMAL1 heterodimer can directly activate the transcription of *PPARα* through the E-box enhancer on its promoter [34], [35]. Interestingly, we found that SHR displayed increased levels of *Bmal1* expression in the heart in parallel with elevated *PPARα* expression during the dark period; changes in feeding cycles affected *Bmal1* expression but did not change *PPARα* expression.

Another possibility is that feeding cycles initially regulate the expression of metabolic genes, which then affect clock gene expression. Among the metabolic genes examined in this study, the PPARs have been shown to directly regulate *Bmal1* expression [22], [26]. Similar to *PPARα*, the expression of *PPARγ* in the heart was elevated during the dark period in SHR. However, in contrast to *PPARα*, the increased levels of *PPARγ* in the heart tissue of SHR were restored to the levels observed in WKY when exposed to an RF regimen. Importantly, we also observed similar effects of RF not only on *Bmal1* but also on *Rev-erbα*, which is another clock gene target of PPARγ [36], suggesting that PPARγ may play an important role in linking metabolic cycles arising from food ingestion with clock gene function in cardiovascular tissues. Indeed, recent work by Wang and coworkers [22] demonstrated that the diurnal BP rhythm is disrupted in mice with a blood vessel-specific deletion of *PPARγ*. Additional evidence that PPARγ coordinates the diurnal rhythm of BP comes from human studies showing that thiazolidinediones, which are PPARγ-selective ligands, are able to normalize the diurnal BP rhythm in diabetic subjects [37].

Our findings that even subtle changes to the feeding cycle can affect diurnal variation in BP and HR raise several important questions. First, what major metabolic signal(s) affect the daily cycle of gene expression in cardiovascular tissues? Among the circulating nutrients and hormones examined in this study, FFA is a strong candidate because only FFA exhibited a disrupted diurnal rhythm in SHR and because this disruption was normalized when SHR were exposed to an RF regimen. Our observation that the diurnal rhythm of lipid metabolism, but not glucose metabolism, was predominantly impaired in SHR supports this idea. For example, SHR displayed attenuated rhythms in lipogenic gene expression in the liver and in fat tissues, whereas the diurnal rhythms of gluconeogenic gene expression were nearly intact in the livers of these animals. Notably, the expression of a large number of lipid-sensing nuclear receptors in the liver, fat, and muscle follows a diurnal rhythm [38]. In addition, Durgan and coworkers [29] demonstrated that the molecular clock in cardiomyocytes is able to sense temporal changes in FFA levels. Combined with these previous reports, our results indicate that the diurnal rhythm of lipid metabolism may play an important role in the generation of circadian rhythms of cardiovascular function.

### Metabolic Cycles and Autonomic Function

Our observation that attenuation of the diurnal rhythm of urine norepinephrine levels in SHR is restored by the correction of feeding cycles indicates a close association between diurnal rhythms of feeding behavior and sympathetic nerve activity. It has been shown that the hypothalamic nuclei involved in feeding behavior have direct neuronal projections to spinal sympathetic preganglionic neurons [39]. Remarkably, in SHR, we observed impaired diurnal rhythms of the expression of neuropeptides in the MBH, suggesting that the attenuation of the norepinephrine rhythm could be due to the dysregulation of feeding circuits in the MBH. On the other hand, strong evidence has established a tight connection between the circadian and sympathetic nerve systems [40], and therefore, peripheral clock gene function can be altered if sympathetic nerve activity is changed. Importantly, the effects of sympathetic nerve activity on the peripheral clock might be tissue-specific; studies using parabiosis between intact and SCN-ablated animals demonstrated that the molecular clock requires neuronal signals to maintain circadian function in the heart, but not in the liver [41]. Indeed, in SHR, we observed that diurnal patterns of *Bmal1* and *Rev-erbα* expression were impaired more severely in the heart than in the liver. Together with previous studies, our results suggest that, in SHR, disrupted rhythms in BP and clock gene expression in the heart could be in part due to altered daily rhythms in sympathetic nerve activity. However, the role of sympathetic input in maintaining the cardiovascular diurnal rhythm remains ambiguous. At the molecular level, although catecholamines affect the phase of clock gene expression in the ex vivo heart [42], the rhythms of clock gene expression in the heart can be entrained to daytime feeding even in catecholamine-deficient mice, which display attenuated BP rhythms [43], [44]. This finding suggests that the sympathetic signal is one of important but not the sole factor that modulates clock gene function in cardiovascular tissues [45]. Further studies should clarify the role of the sympathetic nerve system in mediating the circadian, metabolic, and cardiovascular systems.

### Conclusions

We have shown here that diurnal rhythms of BP and HR are tightly coupled with the daily cycle of feeding behavior in rats with essential hypertension. Furthermore, changes in feeding cycles affect both metabolic and clock gene regulation in cardiovascular tissues. Recent studies have revealed the complex nature of interactions between the circadian and cardiovascular systems [46], as well as between the circadian and metabolic systems [47]. Our observations provide additional evidence that these three systems cooperate to generate diurnal cardiovascular rhythms and suggest that monitoring and/or maintaining the daily cycle of feeding behavior could be beneficial for predicting and improving the prognosis of hypertension, diabetes, and obesity in humans.

## Materials and Methods

### Animals

Male WKY/Izm and SHR/Izm rats were purchased from Japan SLC, Inc. (Hamamatsu, Japan) and maintained in the Laboratory Animal Center at Wakayama Medical University. All experiments were carried out in rats between 8 and 10 weeks of age. All animal care and use procedures were conducted in accordance with guidelines approved by the Wakayama Medical University Institutional Animal Care and Use Committee (Permit Number: 332).

### Blood and Tissue Collection

WKY and SHR (8 weeks old) were maintained under a 12∶12 LD cycle with free access to food and water for 1 week. Animals were then sacrificed at 4 hr intervals across the 24 hr LD cycle. Blood was collected by cardiac puncture. The mediobasal hypothalamus (MBH) was dissected from the brain with two coronal cuts, each just posterior to the optic chiasm and the pituitary stalk. A pair of sagittal cuts was made 2.5 mm from the midline. A final horizontal cut was made 2 mm dorsal to the floor of the MBH. Peripheral tissues (i.e., liver, visceral fat, heart and aorta) were also obtained after the blood and MBH were collected. Serum was separated from whole blood by centrifugation (2,000 x *g*) for 20 min at 4°C and stored at −80°C. Tissues were preserved in RNA stabilization solution at −20°C for subsequent analysis. The specific procedures used to analyze blood and tissue samples are described below.

### Blood Analysis

Serum insulin and leptin concentrations were determined by ELISA (Morinaga Institute of Biological Science, Inc., Yokohama, Japan). Glucose levels were measured with a glucometer (Glutest Neo; Sanwa Kagaku Kenkyusho Co., Ltd., Nagoya, Japan). Free fatty acid (FFA) levels were determined by the enzymatic colorimetric method (NEFA C-test Kit; Wako Diagnostics, Osaka, Japan). Serum corticosterone was analyzed by ELISA (AssayPro, St. Charles, MO).

### RNA Extraction and Quantitative Real-Time PCR

Total RNA was extracted from tissues preserved in RNA stabilization solution with Trizol (Invitrogen, Carlsbad, CA). cDNAs were synthesized using the High Capacity cDNA Reverse Transcription Kit (Applied Biosystems, Foster City, CA). Quantitative real-time PCR was performed and analyzed using a TP850 Thermal Cycler Dice Real-time System (Takara Bio, Inc., Otsu, Japan). Samples contained 1 X SYBR Premix Ex Taq II (Takara Bio, Inc.), 250 nM of each primer, and cDNA in a 10 µl volume. The PCR conditions were as follows: 3 min at 95°C, then 35 cycles of 5 s at 95°C and 20 s at 60°C. Relative expression in comparison with *β-actin* was calculated by the comparative C_T_ method. The sequences of primers used in this study are shown in Table S5.

### Feeding Rhythm Analysis

WKY and SHR (8 weeks old) were caged individually with free access to food and water and maintained under a 12∶12 LD cycle. Food intake was measured immediately before the onset of the dark period and immediately after the onset of the light period. Averages represent intake over 2 consecutive days.

### Analysis of Cardiovascular Parameters

To obtain arterial blood pressure (BP) and heart rate (HR) measurements, a battery-operated transmitter with a pressure-sensing catheter (PA-C40; Data Science International, St Paul, MN) was implanted into 7 week old WKY and SHR. Briefly, the catheter was inserted into the abdominal aorta of animals anesthetized with pentobarbital (50 mg/kg, i.p.), and the transmitter body was placed in the abdominal cavity according to the manufacturer's instructions. Following surgery, animals were given food and water ad libitum and allowed to recover for at least 1 week before data were collected through a receiver (RPC-1; Data Science International). The collected data were stored and analyzed with a computerized system (PowerLab/8s; ADInstruments Japan, Inc., Nagoya, Japan) to determine 24 hr diurnal variation in systolic and diastolic BP and HR. These cardiovascular parameters were monitored for the first 5 min of every 60 min for at least 2 days. Results at each time point represent an average over 2 consecutive days. Both BP and HR were calculated in absolute values and in percent changes from the highest value of each cardiovascular parameter in each individual animal.

### Restricted Feeding Studies

All animals were maintained under a 12∶12 LD cycle and provided with food ad libitum for at least 1 week. One group of animals was then exposed to a restricted feeding (RF) regimen for 5 days, during which food was available only during the dark period while the other group continued to have free access to food. Three sets of animals were used in the RF studies. In the first set of animals, we monitored BP and HR as described above on the fourth and fifth days of the RF regimen. We also measured BP and HR before starting the RF regimen; this measurement served as a baseline condition. In this set of animals, total food intake and BW were also measured on the last day of the RF regimen. In the second set of animals, heart, aorta, and blood were collected every 4 hr on the fifth day of the RF regimen for measurements of serum FFA and gene expression levels. The last set of animals was placed in metabolic cages. After allowing 1 week for acclimatization to the cage, the RF regimen was started, and urine was collected during the light and dark periods on the fourth and fifth days of the RF regimen. We also collected urine for 2 consecutive days before beginning the RF regimen, which served as the baseline condition. Urine was collected in a vial containing 100 µl of 6 M HCl and stored at −20°C for subsequent analysis.

### Catecholamine Assay

Norepinephrine levels in urine were determined by ELISA (GenWay Biotech, Inc., San Diego, CA). The results are shown as average values over the 2 days of urine collection.

### Statistical Analysis

All results are presented as means ± SEM. Two-way analysis of variance (ANOVA) and Bonferroni tests were used for comparisons among multiple groups. A paired or unpaired two-tailed Student's t test was also used for comparisons between two groups. Significance was assumed for p values <0.05.

## Supporting Information

Figure S1 **Effects of restricted feeding on the** ***Cry*** **gene expression in cardiovascular tissues of SHR.** The levels of *Cry1* and *Cry2* mRNA expression in the heart and aorta were determined by real time PCR. Animals were either fed ad libitum or exposed to an RF regimen, in which food was provided exclusively during the dark period, for 5 consecutive days. On the fifth day, heart and aortic tissues were harvested from animals at 4-hr intervals. For visual clarity, only data from ad libitum-fed WKY (black lines, n = 4 per time point) and SHR (red lines, n = 4 per time point) and RF-fed SHR (blue lines, n = 4 per time point) are shown. Values for mRNA expression are displayed as relative expression levels normalized to *β-actin*. The 12∶12 LD cycle is indicated by the bars at the bottom of the figure. All data are expressed as means ± SEM. #, SHR versus WKY; *, SHR fed ad libitum versus RF; p<0.05.
(DOC)

Figure S2 **Effects of restricted feeding on diurnal rhythms of serum free fatty acids levels in SHR.** Animals maintained under a 12∶12 LD cycle were fed ad libitum or maintained under restricted feeding (RF) conditions for 5 consecutive days. Under the RF condition, food was available only during the active (dark) period. On the fifth day of the feeding regimen, blood was collected every 4 hr from the free-fed WKY (black lines, n = 4 per time point) and SHR (red lines, n = 4 per time point) and the RF-fed SHR (blues lines, n = 4 per time point). The levels of free fatty acids (FFA) were determined by the enzymatic colorimetric method. Note that the results for WKY (black lines) and SHR (red lines) fed ad libitum were reproduced from data used in Figure 1C to clarify the difference in diurnal patterns between groups. Values are expressed as means ± SEM.
(DOC)

Table S1 **Food intake and body weight in WKY and SHR.** Food intake (grams) was determined for the 12-hr light, 12-hr dark and total 24-hr periods in the same set of WKY (n = 6) and SHR (n = 6) represented in Figure 1A. Body weight was also compared between these animals. Values are displayed as means ± SEM.
(DOC)

Table S2 **Metabolic parameters in WKY and SHR.** [Note that the data in this table were derived from the same animals represented in Figure 1C.] The levels of glucose, insulin, leptin, and free fatty acids (FFA) in the blood were determined in 8- to 9-week-old animals fed ad libitum. Blood was collected at 4-hr intervals over a 24-hr period from the hearts of WKY (n = 6 per time point) and SHR (n = 6 per time point). The data were pooled to provide an average level of each metabolic parameter over a 24-hr period. Values are presented as means ± SEM.
(DOC)

Table S3 **Cardiovascular parameters in WKY and SHR.** [Note that the data in this table were derived from the same animals represented in Figure 4.] Systolic and diastolic blood pressure (BP) and heart rate (HR) were telemetrically monitored in freely moving WKY (n = 6) and SHR (n = 6). Data were collected for the first 5 min of every 60 min for 2 consecutive days and pooled to determine an overall mean (± SEM) value for the 12-hr light and 12-hr dark periods.
(DOC)

Table S4 **Food intake and body weight in SHR under free and restricted feeding conditions.** SHR were maintained on a 12∶12 LD cycle and exposed either to a free feeding condition or to a restricted feeding (RF) regimen in which food was provided only during the active (dark) period. On the fifth day of the feeding regimens, total food intake over a 24-hr period and body weight were determined in SHR fed ad libitum (n = 6) and SHR exposed to RF (SHR-RF, n = 6). Note that the data in this table were derived from animals 1 to 2 weeks older than those represented in table S1 because the animals in this table were exposed to feeding paradigms for nearly 1 week. All data are expressed as means ± SEM.
(DOC)

Table S5 **Primer sequences used for real-time PCR.**
(DOC)
