# Supplementary material for: Metabolic Cycles Are Linked to the Cardiovascular Diurnal Rhythm in Rats with Essential Hypertension
Source: PLoS One. 2011 Feb 22;6(2):e17339. doi: 10.1371/journal.pone.0017339 (PMC3043102; doi:10.1371/journal.pone.0017339)
Supplement: Figure S1 — Effects of restricted feeding on the Cry gene expression in cardiovascular tissues of SHR. The levels of Cry1 and Cry2 mRNA expression in the heart and aorta were determined by real time PCR. Animals were either fed ad libitum or exposed to an RF regimen, in which food was provided exclusively during the dark period, for 5 consecutive days. On the fifth day, heart and aortic tissues were harvested from animals at 4-hr intervals. For visual clarity, only data from ad libitum-fed WKY (black lines, n = 4 per time point) and SHR (red lines, n = 4 per time point) and RF-fed SHR (blue lines, n = 4 per time point) are shown. Values for mRNA expression are displayed as relative expression levels normalized to β-actin. The 12∶12 LD cycle is indicated by the bars at the bottom of the figure. All data are expressed as means ± SEM. #, SHR versus WKY; *, SHR fed ad libitum versus RF; p<0.05. (DOC) [file pone.0017339.s001.doc]

**Supplemental Data**

**Figure S1**

**Figure S1. Effects of restricted feeding on the *Cry* gene expression in cardiovascular tissues of SHR.** The levels of *Cry1* and *Cry2* mRNA expression in the heart and aorta were determined by real time PCR. Animals were either fed ad libitum or exposed to an RF regimen, in which food was provided exclusively during the dark period, for 5 consecutive days. On the fifth day, heart and aortic tissues were harvested from animals at 4-hr intervals. For visual clarity, only data from ad libitum-fed WKY (black lines, n = 4 per time point) and SHR (red lines, n = 4 per time point) and RF-fed SHR (blue lines, n = 4 per time point) are shown. Values for mRNA expression are displayed as relative expression levels normalized to *β-actin*. The 12:12 LD cycle is indicated by the bars at the bottom of the figure. All data are expressed as means ± SEM. #, SHR versus WKY; *, SHR fed ad libitum versus RF; p < 0.05.
