# Supplementary material for: Metabolic Cycles Are Linked to the Cardiovascular Diurnal Rhythm in Rats with Essential Hypertension
Source: PLoS One. 2011 Feb 22;6(2):e17339. doi: 10.1371/journal.pone.0017339 (PMC3043102; doi:10.1371/journal.pone.0017339)
Supplement: Figure S2 — Effects of restricted feeding on diurnal rhythms of serum free fatty acids levels in SHR. Animals maintained under a 12∶12 LD cycle were fed ad libitum or maintained under restricted feeding (RF) conditions for 5 consecutive days. Under the RF condition, food was available only during the active (dark) period. On the fifth day of the feeding regimen, blood was collected every 4 hr from the free-fed WKY (black lines, n = 4 per time point) and SHR (red lines, n = 4 per time point) and the RF-fed SHR (blues lines, n = 4 per time point). The levels of free fatty acids (FFA) were determined by the enzymatic colorimetric method. Note that the results for WKY (black lines) and SHR (red lines) fed ad libitum were reproduced from data used in Figure 1C to clarify the difference in diurnal patterns between groups. Values are expressed as means ± SEM. (DOC) [file pone.0017339.s002.doc]

**Supplemental Data**

**Figure S2**

**Figure S2. Effects of restricted feeding on diurnal rhythms of serum free fatty acids levels in SHR.** Animals maintained under a 12:12 LD cycle were fed ad libitum or maintained under restricted feeding (RF) conditions for 5 consecutive days. Under the RF condition, food was available only during the active (dark) period. On the fifth day of the feeding regimen, blood was collected every 4 hr from the free-fed WKY (black lines, n = 4 per time point) and SHR (red lines, n = 4 per time point) and the RF-fed SHR (blues lines, n = 4 per time point). The levels of free fatty acids (FFA) were determined by the enzymatic colorimetric method. Note that the results for WKY (black lines) and SHR (red lines) fed ad libitum were reproduced from data used in Figure 1C to clarify the difference in diurnal patterns between groups. Values are expressed as means ± SEM.
