# Supplementary material for: Metabolic Cycles Are Linked to the Cardiovascular Diurnal Rhythm in Rats with Essential Hypertension
Source: PLoS One. 2011 Feb 22;6(2):e17339. doi: 10.1371/journal.pone.0017339 (PMC3043102; doi:10.1371/journal.pone.0017339)
Supplement: Table S1 — Food intake and body weight in WKY and SHR. Food intake (grams) was determined for the 12-hr light, 12-hr dark and total 24-hr periods in the same set of WKY (n = 6) and SHR (n = 6) represented in Figure 1A. Body weight was also compared between these animals. Values are displayed as means ± SEM. (DOC) [file pone.0017339.s003.doc]

**Supplemental Data**

**Table S1. Food intake and body w**eight in WKY and SHR

|  | WKY | SHR | p value |
| --- | --- | --- | --- |
| Food intake (g) |  |  |  |
| Light | 2.1 ± 0.2 | 4.2 ± 0.4 | < 0.01 |
| Dark | 17.2 ± 0.5 | 16.4 ± 0.3 | n.s. |
| Total | 19.3 ± 0.4 | 20.7 ± 0.3 | < 0.05 |
| Body weight (g) | 280.6 ± 4.6 | 261.7 ± 2.6 | < 0.01 |

Food intake (grams) was determined for the 12-hr light, 12-hr dark and total 24-hr periods in the same set of WKY (n = 6) and SHR (n = 6) represented in Figure 1A. Body weight was also compared between these animals. Values are displayed as means ± SEM.
