# Supplementary material for: Metabolic Cycles Are Linked to the Cardiovascular Diurnal Rhythm in Rats with Essential Hypertension
Source: PLoS One. 2011 Feb 22;6(2):e17339. doi: 10.1371/journal.pone.0017339 (PMC3043102; doi:10.1371/journal.pone.0017339)
Supplement: Table S2 — Metabolic parameters in WKY and SHR. [Note that the data in this table were derived from the same animals represented in Figure 1C.] The levels of glucose, insulin, leptin, and free fatty acids (FFA) in the blood were determined in 8- to 9-week-old animals fed ad libitum. Blood was collected at 4-hr intervals over a 24-hr period from the hearts of WKY (n = 6 per time point) and SHR (n = 6 per time point). The data were pooled to provide an average level of each metabolic parameter over a 24-hr period. Values are presented as means ± SEM. (DOC) [file pone.0017339.s004.doc]

**Supplemental Data**

**Table S2. Metabolic p**arameters in WKY and SHR

| Metabolic parameter | WKY | SHR | p *v*alue |
| --- | --- | --- | --- |
| Glucose (mg/dl) | 116.4 ± 5.0 | 124.5 ± 5.4 | n.s. |
| Insulin (ng/ml) | 2.3 ± 0.2 | 2.8 ± 0.2 | n.s. |
| Leptin (ng/ml) | 3.8 ± 0.3 | 3.0 ± 0.2 | < 0.05 |
| FFA (mEq/l) | 0.3 ± 0.1 | 0.6 ± 0.1 | < 0.05 |

[Note that the data in this table were derived from the same animals represented in Figure 1C.] The levels of glucose, insulin, leptin, and free fatty acids (FFA) in the blood were determined in 8- to 9-week-old animals fed ad libitum. Blood was collected at 4-hr intervals over a 24-hr period from the hearts of WKY (n=6 per time point) and SHR (n=6 per time point). The data were pooled to provide an average level of each metabolic parameter over a 24-hr period. Values are presented as means ± SEM.
