# Supplementary material for: Metabolic Cycles Are Linked to the Cardiovascular Diurnal Rhythm in Rats with Essential Hypertension
Source: PLoS One. 2011 Feb 22;6(2):e17339. doi: 10.1371/journal.pone.0017339 (PMC3043102; doi:10.1371/journal.pone.0017339)
Supplement: Table S3 — Cardiovascular parameters in WKY and SHR. [Note that the data in this table were derived from the same animals represented in Figure 4.] Systolic and diastolic blood pressure (BP) and heart rate (HR) were telemetrically monitored in freely moving WKY (n = 6) and SHR (n = 6). Data were collected for the first 5 min of every 60 min for 2 consecutive days and pooled to determine an overall mean (± SEM) value for the 12-hr light and 12-hr dark periods. (DOC) [file pone.0017339.s005.doc]

**Supplemental Data**

**Table S3. Cardiovascular p**arameters in WKY and SHR

|  | WKY | SHR | p value |
| --- | --- | --- | --- |
| Systolic BP (mmHg) |  |  |  |
| Light | 118.4 ± 0.4 | 159.9 ± 0.7 | < 0.001 |
| Dark | 122.9 ± 0.6 | 165.6 ± 0.6 | < 0.001 |
| Diastolic BP (mmHg) |  |  |  |
| Light | 80.8 ± 0.5 | 102.0 ± 0.8 | < 0.001 |
| Dark | 86.4 ± 0.6 | 108.9 ± 0.9 | < 0.001 |
| HR (bpm) |  |  |  |
| Light | 378.1 ± 3.7 | 328.5 ± 3.7 | < 0.001 |
| Dark | 420.3 ± 4.0 | 373.5 ± 3.3 | < 0.001 |

[Note that the data in this table were derived from the same animals represented in Figure 4.] Systolic and diastolic blood pressure (BP) and heart rate (HR) were telemetrically monitored in freely moving WKY (n = 6) and SHR (n = 6). Data were collected for the first 5 min of every 60 min for 2 consecutive days and pooled to determine an overall mean (± SEM) value for the 12-hr light and 12-hr dark periods.
