# Supplementary material for: Metabolic Cycles Are Linked to the Cardiovascular Diurnal Rhythm in Rats with Essential Hypertension
Source: PLoS One. 2011 Feb 22;6(2):e17339. doi: 10.1371/journal.pone.0017339 (PMC3043102; doi:10.1371/journal.pone.0017339)
Supplement: Table S4 — Food intake and body weight in SHR under free and restricted feeding conditions. SHR were maintained on a 12∶12 LD cycle and exposed either to a free feeding condition or to a restricted feeding (RF) regimen in which food was provided only during the active (dark) period. On the fifth day of the feeding regimens, total food intake over a 24-hr period and body weight were determined in SHR fed ad libitum (n = 6) and SHR exposed to RF (SHR-RF, n = 6). Note that the data in this table were derived from animals 1 to 2 weeks older than those represented in table S1 because the animals in this table were exposed to feeding paradigms for nearly 1 week. All data are expressed as means ± SEM. (DOC) [file pone.0017339.s006.doc]

**Supplemental Data**

**Table S4. Food intake and body weight in SHR under free and restricted feeding c**onditions

|  | SHR | SHR-RF | p value |
| --- | --- | --- | --- |
| Food intake (g) | 19.7 ± 0.4 | 20.1 ± 0.4 | n.s. |
| Body weight (g) | 293.8 ± 5.9 | 281.7 ± 4.0 | n.s. |

SHR were maintained on a 12:12 LD cycle and exposed either to a free feeding condition or to a restricted feeding (RF) regimen in which food was provided only during the active (dark) period. On the fifth day of the feeding regimens, total food intake over a 24-hr period and body weight were determined in SHR fed ad libitum (n = 6) and SHR exposed to RF (SHR-RF, n = 6). Note that the data in this table were derived from animals 1 to 2 weeks older than those represented in table S1 because the animals in this table were exposed to feeding paradigms for nearly 1 week. All data are expressed as means ± SEM.
