# Supplementary material for: Metabolic Cycles Are Linked to the Cardiovascular Diurnal Rhythm in Rats with Essential Hypertension
Source: PLoS One. 2011 Feb 22;6(2):e17339. doi: 10.1371/journal.pone.0017339 (PMC3043102; doi:10.1371/journal.pone.0017339)
Supplement: Table S5 — Primer sequences used for real-time PCR. (DOC) [file pone.0017339.s007.doc]

**Supplemental Data**

**Table S5. Primer sequences used for real-time PCR**

| Gene name | Sequences |
| --- | --- |
| *Bmal1* | 5`-GACAATGAGGGCTGCAATCT-3` |
|  | 5`-GTGGAACCATGTGTGAGTGC-3` |
| *Clock* | 5`-CACATGATCCAGCAGCAGAC-3` |
|  | 5`-TTGGCTGACTGTGTCCACTC-3` |
| *Per2* | 5`-CCCACACTTGCCTCTGAAAT-3` |
|  | 5`-CGAGGTCCGACTAGGGAACT-3` |
| *Rev-erbα* | 5`-CAGCTTCCAGTCCCTGACTC-3` |
|  | 5`-GTGAGGGAGCCAGTAGGTGA-3` |
| *Cry1* | 5`-ACCCGCGGCGACCTATGGAT-3` |
|  | 5`-GCATTGATGCTCCAGTCGGCGT-3` |
| *Cry2* | 5`-AGGCTTCCCTTGGATTGACGCC-3` |
|  | 5`-TGAGGAAGCAGGCCACAGCG-3` |
| *POMC* | 5`-GCTTCATGACCTCCGAGAAG-3` |
|  | 5`-TCTTGATGATGGCGTTCTTG-3` |
| *NPY* | 5`-TACTCCGCTCTGCGACACTA-3` |
|  | 5`-TGTCTCAGGGCTGGATCTCT-3` |
| *AgRP* | 5`-CCCTGTTCCCAGAGTTCTCA-3` |
|  | 5`-ACATCTTCTGCTCGGTCTGC-3` |
| *SREBP-1c* | 5`-GGAGCCATGGATTGCACATTTG-3` |
|  | 5`-CAAATAGGCCAGGGAAGTCAC-3` |
| *ACC* | 5`-CATCAGGCACTCTGATCTGG-3` |
|  | 5`-GAGCAGTCGCTCTCCTTCAT-3` |
| *FAS* | 5`-AAGAGCATTCTGGCCACATC-3` |
|  | 5`-TTCATGGCTGTTAGCCACAC-3` |
| *SCD-1* | 5`-CAGTTCCTACACGACCACCA-3` |
|  | 5`-AGGGGCACCTTCTTCATCTT-3` |
| *PEPCK* | 5`-ACGCCATTAAGACCATCCAG-3` |
|  | 5`-ACCCCCATCACTTGTCTCAG-3` |
| *G6P* | 5`-GTTTGGTTTCGCACTTGGAT-3` |
|  | 5`-TCCAAAGTCCACAGGAGGTC-3` |
| *PPARα* | 5`-TTGTGCATGGCTGAGAAGAC-3` |
|  | 5`-ACTGGCAGCAGTGGAAGAAT-3` |
| *PPARγ* | 5`-ATGGGTGAAACTCTGGGAGA-3` |
|  | 5`-GCTCATAGGCAGTGCATCAG-3` |
| *β-actin* | 5`-GTCGTACCACTGGCATTGTG-3` |
|  | 5`-ACCCTCATAGATGGGCACAG-3` |
